# Constitutive expression of the deubiquitinating enzyme CYLD does not affect microglia phenotype or function in homeostasis and neuroinflammation

**DOI:** 10.1007/s00109-024-02489-7

**Published:** 2024-09-20

**Authors:** Eva Schramm, Vanessa Becker, Ilaria Palagi, Melanie Müller, Thomas Rösler, Feyza Durak, Anna Ebering, Khalad Karram, Esther von Stebut, Michael J. Schmeisser, Ari Waisman

**Affiliations:** 1 Institute for Molecular Medicine, University Medical Center of the Johannes Gutenberg-University Mainz, Langenbeckstraße 1, 55131 Mainz, Germany; 2 TRON–Translational Oncology at the University Medical Center of Johannes Gutenberg University Mainz gGmbH, Mainz, Germany; 3 Department of Dermatology, University of Cologne, Cologne, Germany; 4 Institute of Anatomy, University Medical Center of the Johannes Gutenberg-University Mainz, Mainz, Germany; 5 Focus Program Translational Neurosciences (FTN), University Medical Center of the Johannes Gutenberg-University Mainz, Mainz, Germany; 6 Research Center for Immunotherapy (FZI), University Medical Center of the Johannes Gutenberg-University Mainz, Mainz, Germany

**Keywords:** CYLD, Deubiquitinating enzyme, Microglia, Neuroinflammation, NF-κB, EAE

## Abstract

**Abstract:**

The deubiquitinating enzyme CYLD negatively regulates NF-κB signaling by removing activating ubiquitin chains from several members of the NF-κB pathway. Thereby, CYLD is critical for the maintenance and differentiation of various immune cells. Despite the importance of the NF-κB pathway in microglia regulation, the role of CYLD in microglia has not been investigated so far. In this study, we investigated whether CYLD in microglia can protect against neuroinflammation using a newly generated conditional mouse strain (Rosa26-Cyld-tdTomato) that allows cell type-specific CYLD overexpression. Here, we show that overexpression of CYLD in microglia did not alter microglia numbers or microglia morphology in different brain regions. Additionally, CYLD overexpression did not modify the microglial response to LPS-induced neuroinflammation or the disease severity in experimental autoimmune encephalomyelitis (EAE). Finally, also immune cell infiltration into the CNS during EAE and under steady state conditions remained unaffected by microglial CYLD overexpression. Our findings suggest that CYLD overexpression does not alter microglial function, and thus does not represent a viable therapeutic strategy in neuroinflammatory conditions. This study highlights the complexity of ubiquitin-mediated signaling in neuroinflammation and the need for cell-type-specific investigations. The Rosa26-Cyld-tdTomato mouse model offers a valuable tool for studying CYLD’s role across various tissues and cell types.

**Key messages:**

- Novel mouse strain for cell type-specific overexpression of the deubiquitinating enzyme CYLD.
- CYLD overexpression in microglia did not alter microglia numbers or morphology in the steady state.
- CYLD overexpression in microglia did not protect mice from LPS-induced neuroinflammation or EAE.
- CYLD overexpression in microglia did not influence their gene expression during neuroinflammation.

## Introduction

The deubiquitinating enzyme CYLD was first identified as a tumor suppressor gene mutated in familial cylindromatosis, a genetic condition predisposing individuals to develop multiple skin tumors [1, 2]. Located in the cytoplasm, CYLD removes Lysine 63 (K63)- or Met1-linked polyubiquitin chains from several substrates [3]. Among these substrates are molecules of the proinflammatory NF-κB pathway such as TRAF2, TRAF6, and NEMO, and CYLD acts as a negative regulator of NF-κB signaling by removing activating K63-linked ubiquitin chains from these proteins [4–6]. By controlling NF-κB pathway activity, CYLD regulates the maintenance and differentiation of various immune cell types. Studies with CYLD-deficient mice showed that CYLD is required for the proper development and activation of T and B cells [7–9]. Previously, we have generated mice deficient in full-length CYLD but overexpressing a naturally occurring splice variant of CYLD that lacks the binding sites for TRAF2 and NEMO [10]. These CYLD^ex7/8^ mice show defects in B cell homeostasis [10] and regulatory T cell function [11], and exhibit a hyperactive phenotype of dendritic cells and T cells [11–13].

CYLD is expressed in the brain [2] and is required for maintaining neuronal activity and synaptic transmission [14, 15]. Recent studies showed that CYLD deficiency leads to an autistic phenotype characterized by impaired sociability and other behavioral abnormalities, likely due to the loss of CYLD’s regulation of mTOR signaling and autophagy [16, 17].

Microglia are innate immune cells residing in the central nervous system (CNS) parenchyma. In the steady state, microglia have a ramified morphology with finely branched processes that scan the CNS environment for potential threats [18]. Upon infection or disease, microglia adopt an amoeboid shape, become reactive and phagocytose cellular debris, dead cells and foreign material. In studies with *Cyld*^−/−^ mice, microglia seem to exhibit a more activated phenotype in certain brain regions [14, 19]. A similar phenotype was observed in mice lacking another deubiquitinating enzyme that negatively regulates the NF-κB pathway, namely A20, specifically in microglia [20, 21]. Mice with A20 deletion in microglia exhibited increased microglia numbers and altered microglia morphology, suffered from spontaneous neuroinflammation and showed exacerbated responses to different mouse models of neuroinflammation [20, 21]. Interestingly, while the protein substrates of A20 and CYLD partially overlap, mice deficient in one of the enzymes nevertheless display different phenotypes [22].

We hypothesized that mice with CYLD-deficient microglia could also be more prone to develop neuroinflammation, while constitutive overexpression of CYLD in microglia could protect against CNS inflammation. To study the cell-type specific role of CYLD in microglia, we generated a novel mouse strain allowing the conditional overexpression of CYLD in a cell type of interest (Rosa26-Cyld-tdTomato mice) and used these mice to study the effects of constitutive CYLD expression in microglia. Surprisingly, microglia numbers and microglia morphology were not altered in several brain regions of mice overexpressing CYLD in microglia. Additionally, constitutive CYLD expression in microglia did not affect CNS immune cell infiltration either in the steady state or in experimental autoimmune encephalomyelitis (EAE), a mouse model for Multiple Sclerosis (MS). Even when directly stimulating the NF-κB pathway in microglia in a model of LPS-induced neuroinflammation, CYLD overexpression had no effect on microglia gene expression and function. Taken together, our data strongly suggest that constitutive expression of CYLD in microglia does not impact microglia function and is not protective in the context of neuroinflammation.

## Results

### Overexpression of Cyld in microglia does not influence microglia numbers and microglia morphology in the steady state

**Fig. 1:**
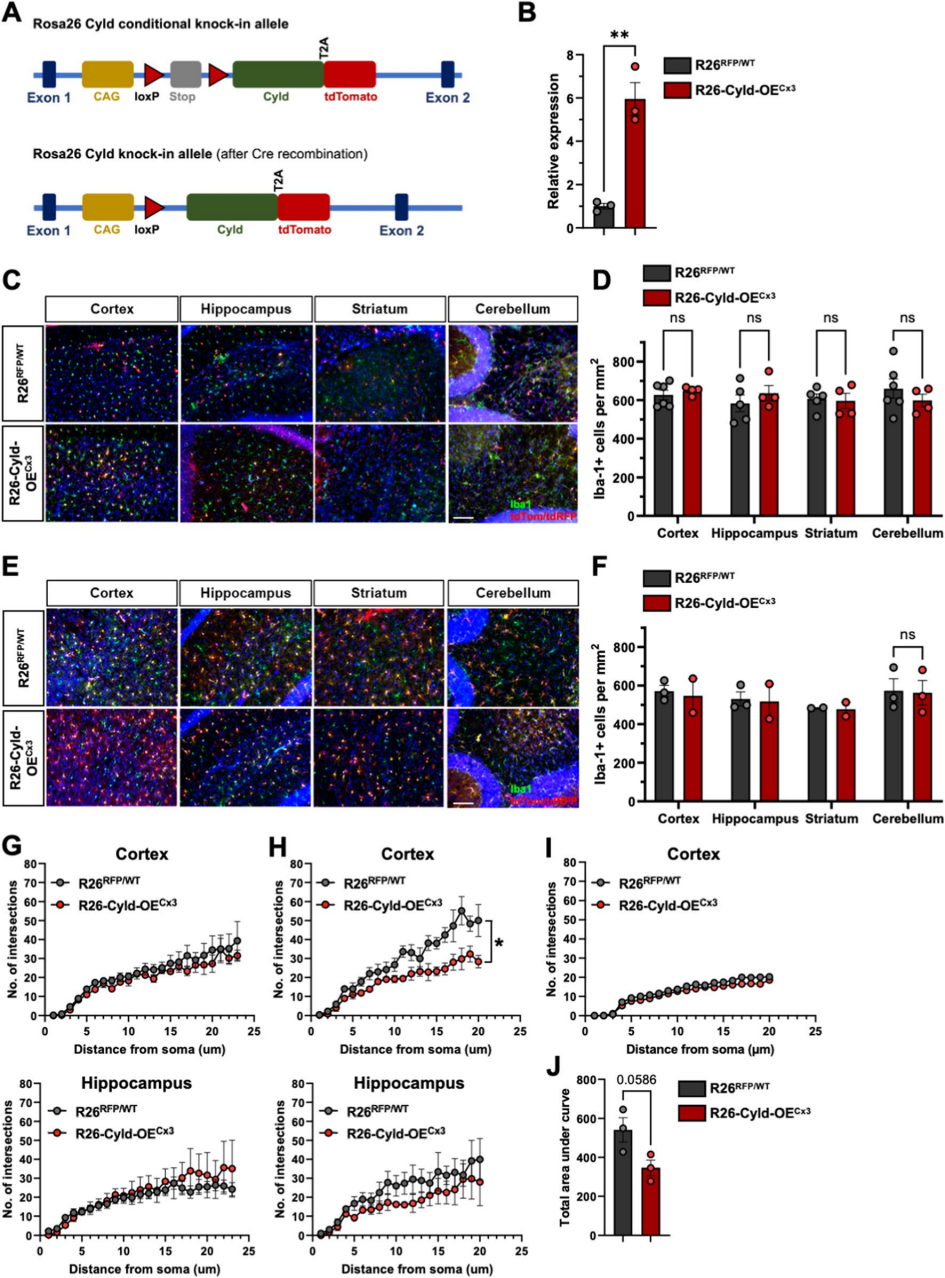
Overexpression of *Cyld* in microglia does not influence microglia numbers and microglia morphology in the steady state. **A** Rosa26 Cyld conditional knock-in mice were generated in which the *Cyld* gene and the *tdTomato* gene were inserted into the Rosa26 locus behind a STOP cassette flanked by loxP sites (top). After Cre-mediated removal of the STOP cassette, the constitutive knock-in allele is obtained (bottom). **B** Expression of *Cyld* in primary murine microglia isolated by magnetic-activated cell sorting (MACS) for CD11b was validated by RT-PCR. *Cyld* mRNA levels were normalized to *Hprt* and plotted relative to control. **C** Representative images of tdTomato/tdRFP-labelled Iba-1^+^ (green) microglia in different brain regions of R26-Cyld-OE^Cx3^ mice and littermate controls 2 weeks after tamoxifen administration. Scale bar 100 μm. **D** Quantification of Iba-1^+^ cells per mm^2^ 2 weeks after tamoxifen administration. **E** Representative images of tdTomato/tdRFP-labelled Iba-1^+^ (green) microglia in different brain regions of R26-Cyld-OE^Cx3^ mice and littermate controls 12 weeks after tamoxifen administration. Scale bar 100 μm. **F** Quantification of Iba-1^+^ cells per mm^2^ 12 weeks after tamoxifen administration. **G** Sholl analysis of microglia in the cortex and hippocampus of R26-Cyld-OE^Cx3^ mice and littermate controls 2 weeks after tamoxifen administration. N = 6 mice for R26^RFP/WT^ controls; N = 4 mice for R26-Cyld-OE^Cx3^; n = 9–17 cells. **H** Sholl analysis of microglia in the cortex and hippocampus of R26-Cyld-OE^Cx3^ mice and littermate controls 12 weeks after tamoxifen administration. N = 3 mice for R26^RFP/WT^ controls; N = 3 mice for R26-Cyld-OE^Cx3^; n = 4–9 cells. **I** Sholl analysis of microglia in the cortex of R26-Cyld-OE^Cx3^ mice and littermate controls 6 months after tamoxifen administration. N = 4 mice for R26^RFP/WT^ controls; N = 4 mice for R26-Cyld-OE^Cx3^; n = 36 cells. **J** Area under curve of the Sholl analysis of the cortex 12 weeks after tamoxifen administration shown in (**H**). In **B**, **D**, **F**, and **J**, every circle represents a single mouse. Data is represented as mean ± SEM. Statistical significance was determined by two-tailed unpaired Student’s t-test (**B**, **D**, **F**, **J**) or two-way ANOVA with Bonferroni Multiple comparisons correction (**G**, **H**, **I**). * p < 0.05, ** p < 0.01, **** p < 0.0001, ns = non-significant

To assess the role and function of CYLD in microglia, a new conditional mouse strain was generated in which CYLD can be overexpressed in specific cell types in a Cre-dependent manner. These Rosa26-Cyld-tdTomato mice were generated by insertion of the *Cyld* and *tdTomato* genes into the Rosa26 locus behind a loxP-flanked STOP cassette (Fig. 1A). After Cre-mediated removal of the STOP cassette, a chimeric transcript of *Cyld* and *tdTomato* fused by a T2A site is expressed. The T2A sequence leads to co-translational cleavage between the CYLD and tdTomato proteins allowing the co-expression of CYLD and tdTomato in the cell type of interest. We crossed these Rosa26-Cyld-tdTomato mice to the tamoxifen-inducible Cx3cr1^CreER^ strain [23] to obtain R26-Cyld-OE^Cx3^ mice. In the CNS of R26-Cyld-OE^Cx3^ mice in the steady state, the expression of CYLD and tdTomato is limited to microglia and other CNS-resident macrophage populations (for simplicity, we will only use the term "microglia" in the following sections). We used littermate control animals for all experiments, which harbored an allele of the *tdRFP* reporter gene in the Rosa26 locus [24] but no *Cyld* (R26^RFP/WT^), and also expressed the tamoxifen-inducible Cre recombinase under control of the *Cx3Cr1* promotor. We verified the expression of *Cyld* in isolated primary microglia of R26-Cyld-OE^Cx3^ mice where we observed on average 5.95-fold higher expression levels of *Cyld* than in R26^RFP/WT^ controls (Fig. 1B). Next, we quantified microglia numbers in the motor cortex, hippocampus, striatum and cerebellum of R26-Cyld-OE^Cx3^ mice and controls two weeks (Fig. 1C, D) and twelve weeks (Fig. 1E, F) after tamoxifen administration. CYLD overexpression in R26-Cyld-OE^Cx3^ mice had no effect on microglia numbers in the brain regions assessed. We also performed a Sholl analysis in the motor cortex and hippocampus to assess the morphology of the microglia. Microglia branching complexity was not altered in R26-Cyld-OE^Cx3^ mice in the motor cortex and hippocampus two weeks after tamoxifen administration (Fig. 1G), as well as in the hippocampus twelve weeks after tamoxifen administration (Fig. 1H). Surprisingly, microglia of R26-Cyld-OE^Cx3^ mice showed a slightly decreased branching complexity with less intersections in the motor cortex twelve weeks after tamoxifen administration (Fig. 1H, J). To further investigate this phenotype, we also performed a Sholl analysis in the motor cortex six months after tamoxifen administration. At this timepoint, microglia branching complexity was not altered in R26-Cyld-OE^Cx3^ mice compared to R26^RFP/WT^ littermate controls (Fig. 1I).

### Overexpression of Cyld in microglia does not influence immune cell infiltration and microglia phenotype in the steady state

**Fig. 2:**
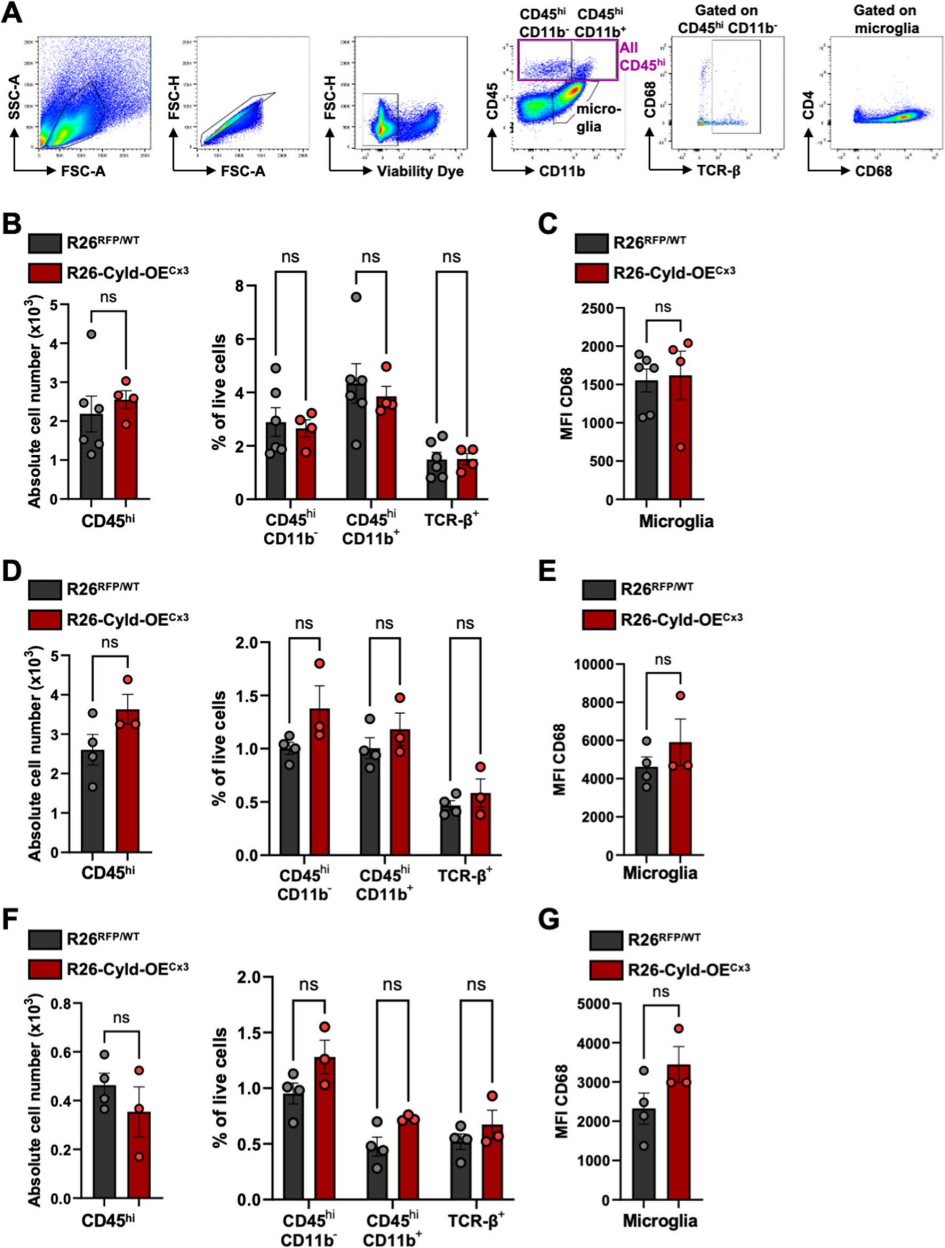
Overexpression of *Cyld* in microglia does not influence immune cell infiltration and microglia phenotype in the steady state. **A** Representative gating strategy for the flow cytometry analysis of immune cells and microglia isolated from the CNS. **B** Quantification of the absolute cell number of CD45^hi^ immune cells in the brain (left) and percentages of CD45^hi^ CD11b^−^, CD45^hi^ CD11b^+^ and TCR-β^+^ cells among all brain cells (right) 2 weeks after tamoxifen administration. **C** Mean fluorescence intensity (MFI) as geometric mean of CD68 on brain microglia 2 weeks after tamoxifen administration. **D** Quantification of the absolute cell number of CD45^hi^ immune cells in the brain (left) and percentages of CD45^hi^ CD11b^−^, CD45^hi^ CD11b^+^ and TCR-β^+^ cells among all brain cells (right) 12 weeks after tamoxifen administration. **E** Mean fluorescence intensity (MFI) as geometric mean of CD68 on brain microglia 12 weeks after tamoxifen administration. **F** Quantification of the absolute cell number of CD45^hi^ immune cells in the spinal cord (left) and percentages of CD45^hi^ CD11b^−^, CD45^hi^ CD11b^+^ and TCR-β^+^ cells among all spinal cord cells (right) 12 weeks after tamoxifen administration. **G** Mean fluorescence intensity (MFI) as geometric mean of CD68 on spinal cord microglia 12 weeks after tamoxifen administration. Every circle represents a single mouse. Data is represented as mean ± SEM. Statistical significance was determined by two-tailed unpaired Student’s t-test. ns = non-significant

Since mice lacking A20 in microglia developed spontaneous neuroinflammation with an increased infiltration of CD8^+^ T cells into the CNS of these mice [21], we hypothesized that mice overexpressing CYLD in microglia might show an opposite phenotype. Therefore, we analyzed immune cell infiltrates in the brain and spinal cord at steady state by flow cytometry (Fig. 2A). No alterations in the number of CD45^hi^ immune cells in the brains of R26-Cyld-OE^Cx3^ mice compared to controls were detected two weeks after tamoxifen administration. Similarly, also no changes in the percentages of CD45^hi^ CD11b^−^ infiltrates, CD45^hi^ CD11b^+^ infiltrates, and TCR-β^+^ T cells among all brain cells could be observed (Fig. 2B). Of note, at this early time point after tamoxifen administration, also short-lived Cx3Cr1^+^ cells such as peripheral monocytes could harbor the Rosa26-Cyld-tdTomato allele and therefore might overexpress CYLD. Next, we analyzed the brain and spinal cord twelve weeks after tamoxifen administration. Also here, no differences in the numbers and percentages of immune cells were observed in R26-Cyld-OE^Cx3^ mice compared to littermate controls (Fig. 2D, F). Finally, we also examined expression levels of CD68, a lysosomal marker upregulated in phagocytically active microglia. No alterations in the mean fluorescence intensity (MFI) of CD68 in microglia could be observed in R26-Cyld-OE^Cx3^ mice compared to controls in the brain and spinal cord at the respective time points (Fig. 2 C, E, G). Therefore, we concluded that CYLD overexpression in microglia does not affect immune cell infiltration in the steady state.

### Conditional overexpression of Cyld in microglia and Cyld deletion do not affect EAE pathology

**Fig. 3:**
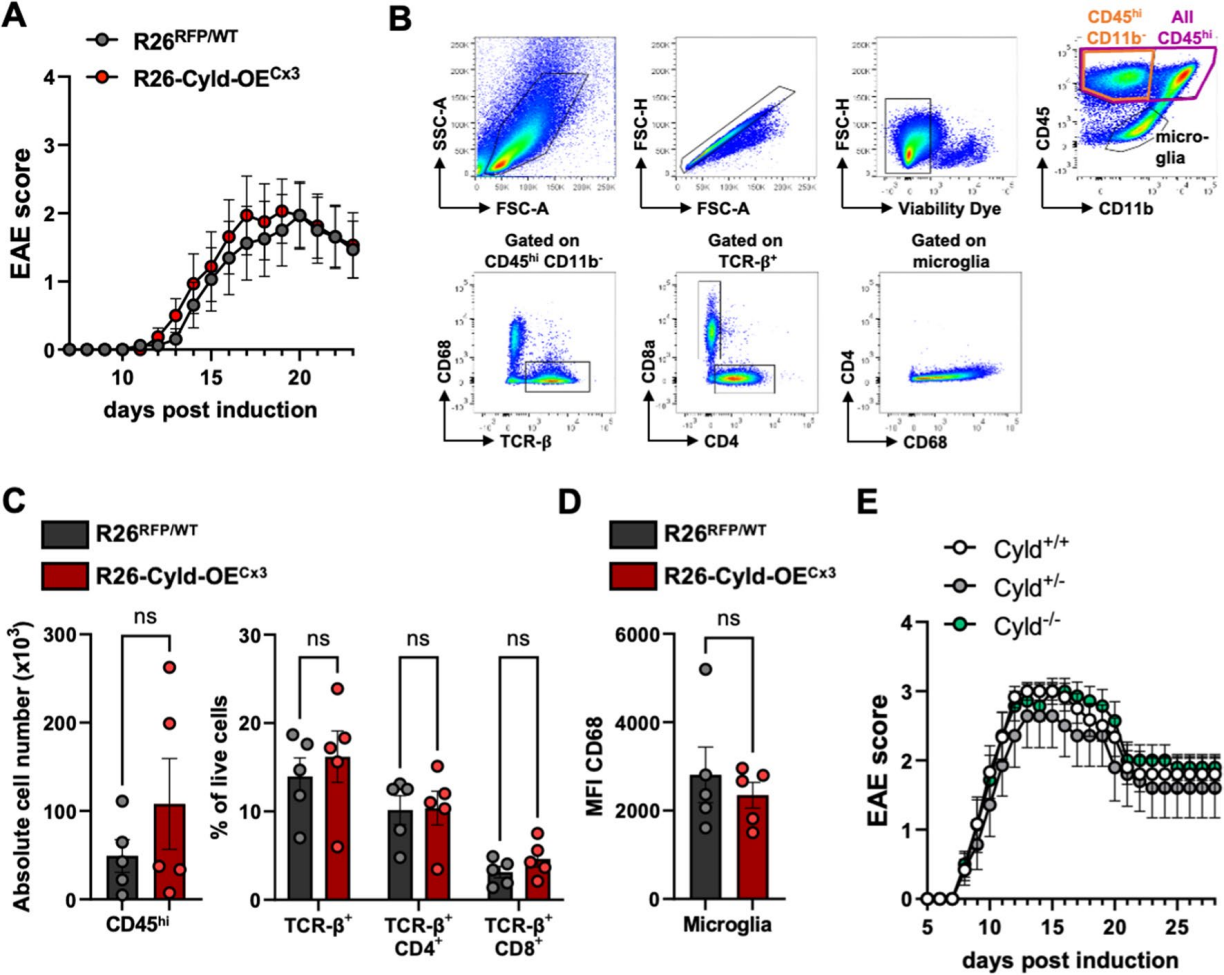
Conditional overexpression of *Cyld* in microglia and *Cyld* deletion do not affect EAE pathology. **A** EAE was induced in R26-Cyld-OE^Cx3^ mice and littermate controls and clinical signs of EAE were monitored daily. Data is pooled from two independent experiments with a total of n = 8 mice per group. **B** Representative gating strategy for the flow cytometry analysis of immune cells and microglia isolated from the spinal cord of EAE mice at day 23 after EAE induction. **C** Quantification of the absolute cell number of CD45^hi^ immune cells in the spinal cord (left) and percentages of CD45^hi^ CD11b^−^, CD45^hi^ CD11b^+^ and TCR-β^+^ cells among all spinal cord cells (right). **D** Mean fluorescence intensity (MFI) as geometric mean of CD68 on spinal cord microglia. **E** EAE was induced in *Cyld*^−/−^ mice (n = 7) and Cyld^+/-^ (n = 6) and *Cyld*^+/+^ (n = 6) littermate controls. Clinical signs of EAE were monitored daily. In **C** and **D**, every circle represents a single mouse. Data is represented as mean ± SEM. Statistical significance was determined by two-tailed unpaired Student’s t-test (**C**, **D**). ns = non-significant

Since immune cell infiltration into the CNS in the steady state is very limited, the potential role of CYLD in microglia as a negative regulator of neuroinflammation may only be required during neuroinflammatory challenges. Therefore, we immunized R26-Cyld-OE^Cx3^ mice and controls with the MOG_35-55_ peptide to induce experimental autoimmune encephalomyelitis (EAE) and monitored clinical signs of disease. We observed no effect of CYLD overexpression in microglia on EAE disease progression (Fig. 3A). We also isolated spinal cord cells of the EAE mice at day 23 after induction of EAE for flow cytometry analysis (Fig. 3B). As was already expected with regard to the similar EAE scores, no differences in immune cell infiltrates could be observed between R26-Cyld-OE^Cx3^ mice and littermate controls (Fig. 3C). Also the expression levels of CD68 on microglia did not differ between R26-Cyld-OE^Cx3^ mice and controls, indicating no influence of CYLD overexpression on microglia activation during EAE (Fig. 3D). We also wanted to analyze the effect of *Cyld* deletion on the development of EAE and immunized Cyld^−/−^ mice [25] and their heterozygous (Cyld^+/-^) and homozygous (Cyld^+/+^) littermate controls with the MOG_35-55_ peptide to induce EAE. Also the full knock-out of *Cyld* had no effect on EAE disease progression (Fig. 3E) questioning the importance of CYLD in microglia as a regulator of autoimmune neuroinflammation.

### Overexpression of Cyld in microglia does not alter the microglial response to lipopolysaccharide (LPS)

**Fig. 4:**
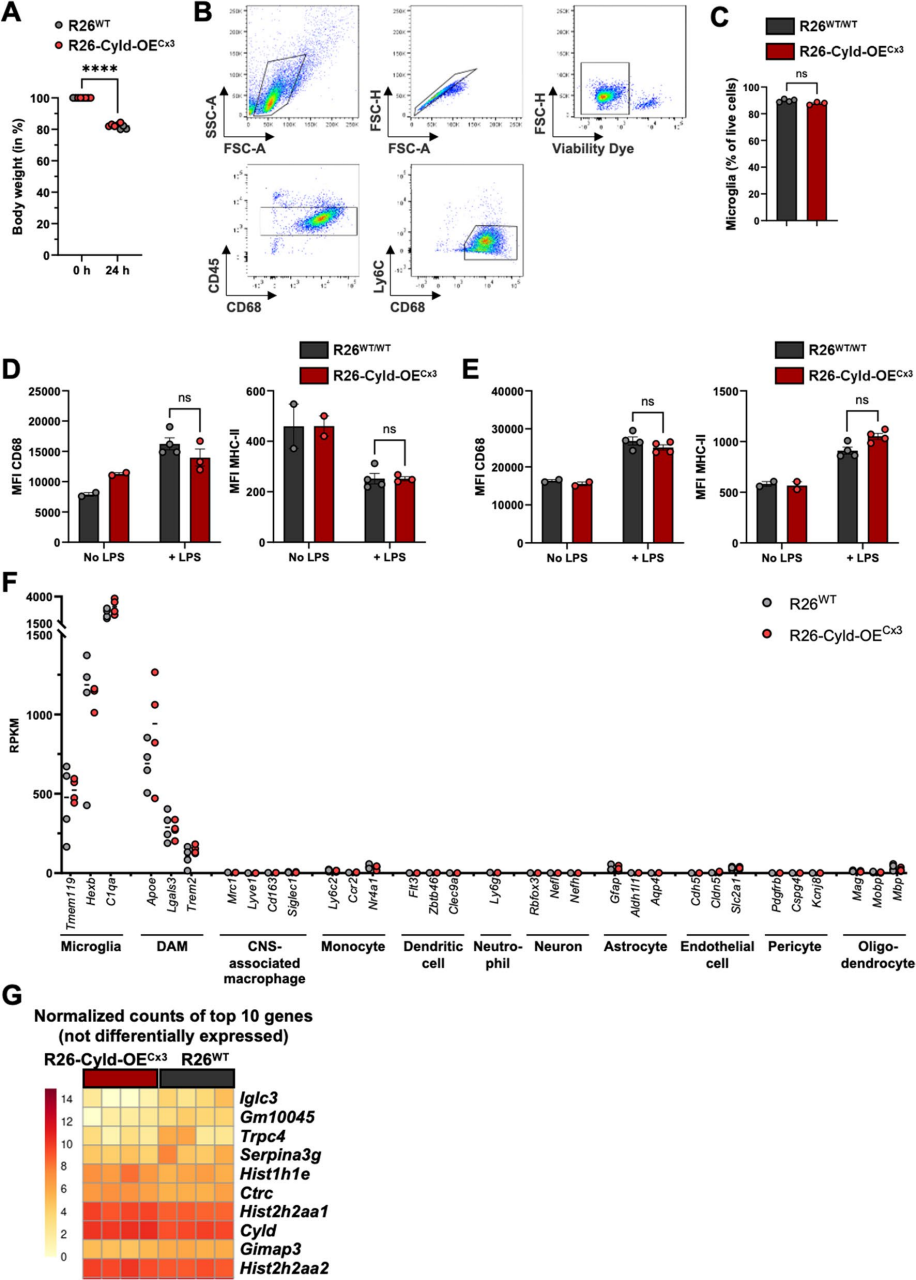
Overexpression of* Cyld* in microglia does not alter the microglial response to LPS. R26-Cyld-OE^Cx3^ mice and littermate controls were injected intraperitoneally with 1 mg/kg LPS and microglia were isolated for flow cytometry and RNA sequencing 24 h later. **A **Change in body weight 24 h after LPS injection (shown as % of starting body weight). **B** Representative gating strategy for the flow cytometry analysis of brain and spinal cord microglia (brain is shown). From brain CNS samples, microglia were first isolated by MACS for CD11b^+^ cells. For spinal cord samples, the whole CNS cell suspension was used for flow cytometry. After gating on live, single cells, microglia were selected as CD45^intermediate^, CD68^+^ and Ly6C^−^. **C** Microglia purity was analyzed by flow cytometry in the MACS-isolated brain samples used for RNA sequencing. **D** Mean fluorescence intensity (MFI) as geometric mean of CD68 and MHC-II on brain microglia. **E** Mean fluorescence intensity (MFI) as geometric mean of CD68 and MHC-II on spinal cord microglia. **F** Purity of MACS-isolated microglia was confirmed by plotting reads per kilobase of transcript per million mapped reads (RPKM) for marker genes of CNS and immune cell types. **G** Normalized counts of the top 10 genes with the greatest difference in expression between R26-Cyld-OE^Cx3^ and control microglia (not differentially expressed). In **A**, **C**, **D** and **E**, every circle represents a single mouse. Data in **A**, **C**, **D** and **E** is represented as mean ± SEM. Statistical significance was determined by two-tailed unpaired Student’s t-test (**A**, **C**, **D**, **E**). ***** p < 0.0001, ns = non-significant

In addition to the EAE model in which CYLD overexpression in microglia did not confer protection against autoimmune neuroinflammation in R26-Cyld-OE^Cx3^ mice, we used a model of LPS-induced neuroinflammation to directly stimulate microglial inflammatory responses. LPS activates microglia and induces the production of proinflammatory cytokines in the brain [26] by binding to toll-like receptor 4 (TLR4) on microglia [27], which activates the NF-κB pathway. We injected R26-Cyld-OE^Cx3^ mice and controls intraperitoneally with 1 mg/kg LPS and isolated their microglia for flow cytometry and RNA sequencing 24 h later. All mice showed a significant decrease in body weight 24 h after LPS injection confirming that an inflammatory response was triggered (Fig. 4A). Microglia were isolated by MACS for CD11b^+^ cells and purity was confirmed by flow cytometry (Fig. 4B), with microglia accounting for 87–91% of all live cells in the samples isolated for RNA sequencing (Fig. 4C). No differences in microglia activation could be observed in flow cytometry with similar expression levels of CD68 and MHC-II on brain microglia (Fig. 4D) and spinal cord microglia (Fig. 4E) of R26-Cyld-OE^Cx3^ mice and littermate controls. The RNA sequencing data were analyzed for signature genes of different CNS and immune cell types to further confirm the purity of the samples. Microglia signature genes *Tmem119*, *Hexb* and *C1qa* were highly expressed, while marker genes of neurons, astrocytes, endothelial cells, pericytes and oligodendrocytes were barely detectable (Fig. 4F). Also marker genes of other immune cell types such as monocytes, dendritic cells and neutrophils, as well as marker genes of CNS-associated macrophages such as *Mrc1* and *Lyve1* were absent. Interestingly, genes known to be expressed by “disease-associated microglia” (DAMs) [28], namely *Apoe*, *Lgals3* and *Trem2*, were found to be expressed by the microglia. We also confirmed that LPS treatment efficiently activated the microglia by comparing the gene expression profile of naïve *versus* LPS-treated microglia, where we observed 3001 differentially expressed genes (1471 upregulated; 1530 downregulated; threshold: adjusted p-value < 0.05, log2 fold change > 1; data not shown). Next, we performed a DESeq2 analysis for differentially expressed genes in LPS-treated R26-Cyld-OE^Cx3^ *versus* LPS-treated R26^RFP/WT^ microglia samples (threshold: adjusted p-value < 0.05, log2 fold change > 1). Figure 4G shows the 10 genes with the greatest difference in expression between the two groups. However, surprisingly, we could not detect any statistically significant DEGs. This rather unexpected finding strongly supports our previous results and suggests the conclusion that CYLD overexpression in microglia has no effect on microglia gene expression and function in the context of neuroinflammation.

## Discussion

CYLD inhibits NF-κB activation, which is a key pathway in regulating microglia during inflammation and in various CNS diseases [29]. However, the specific mechanisms by which NF-κB is regulated in microglia and the potential pathways targeted by CYLD beyond NF-κB remain incompletely understood. Given the detrimental effects of A20 deletion in microglia [20, 21], an enzyme that functions similar to CYLD, we hypothesized that CYLD overexpression in microglia could have the opposite effect and might protect mice against CNS inflammation. Contrary to our expectations, overexpression of CYLD in microglia did not affect microglial gene expression or function in LPS-induced neuroinflammation and did not influence disease severity in the EAE model. Additionally, in the steady state, CYLD overexpression had no effect on immune cell infiltration into the CNS or on microglia numbers in different brain regions. Thus, we conclude that constitutive overexpression of CYLD in microglia cannot serve as a strategy to protect mice from neuroinflammation.

Our most compelling evidence for this conclusion comes from the model of LPS-induced neuroinflammation. LPS is the most common stimulus for microglia activation in vivo and in vitro [30] and acts by activating the NF-κB pathway in microglia via the activation of TLR4. We induced the overexpression of CYLD in microglia by tamoxifen injection and analyzed the microglial response to LPS 12 weeks later. To our surprise, no differences between R26-Cyld-OE^Cx3^ mice and R26^RFP/WT^ littermate controls could be observed on the protein and gene expression levels, despite clear expression changes between naïve and LPS-treated mice, which confirmed that our analysis approach in general worked. This was further corroborated in the EAE model, where CYLD overexpression in microglia did not influence disease severity or immune cell infiltration into the CNS.

In contrast, deletion of the deubiquitinating enzyme A20 in microglia significantly enhanced microglia activation and neuroinflammation. Mice with A20 deletion were more susceptible to LPS and EAE and exhibited a hyperactive microglial phenotype with increased proinflammatory cytokine production [20]. One possible explanation for our observation that microglial CYLD overexpression had no effect, while microglial A20 deletion was detrimental, is that even though CYLD and A20 share a number of substrates, they also have distinct substrate specificities and A20 has non-catalytic functions that CYLD lacks [22]. Furthermore, while mice with full deletion of A20 suffer from multiorgan inflammation and die at a young age [31], the loss of CYLD is not problematic [25] indicating that A20 is more critical in preventing excessive immune responses. This could be due to the fact that A20 is upregulated in a NF-κB-dependent manner which serves as a negative feedback mechanism to terminate NF-κB signaling [32]. Another factor could be that CYLD is expressed at much lower levels in microglia compared to A20 [33], potentially limiting its role in regulating microglia function under physiological conditions. However, even if CYLD is physiologically not required for microglia function, we hypothesized that its overexpression could nonetheless protect against neuroinflammation by restraining NF-κB signaling. One hypothesis for the lack of this protective effect is that the artificially expressed CYLD could be functionally inactivated, possibly through phosphorylation or cleavage [22]. In conclusion, we are convinced that CYLD overexpression in microglia is not effective to protect mice from neuroinflammation. However, we cannot rule out the possibility that endogenous CYLD in microglia could play a role in the regulation of neuroinflammation. Further studies with conditional CYLD deletion in microglia are needed to explore this possibility.

Also in the steady state, CYLD overexpression in microglia did not affect microglia numbers across different brain regions or the infiltration of peripheral immune cells into the CNS. In contrast, mice with A20 deletion in microglia showed increased microglia numbers [20, 21] and spontaneous infiltration of CD8^+^ T cells in the CNS [21], again suggesting that A20 is more critical for the regulation of microglia physiology. We observed a slight change in microglia morphology in the cortex of R26-Cyld-OE^Cx3^ mice twelve weeks post-tamoxifen injection, but this was not seen at two weeks or six months, questioning the significance of this finding. While no studies with a microglia-specific *Cyld* deletion have been conducted so far, global *Cyld*^−/−^ mice displayed altered microglial numbers and morphology, along with elevated levels of the proinflammatory cytokines IL-1β and TNF-α in the dorsal striatum, indicating a more activated microglia phenotype [14, 19]. This may result from the loss of CYLD in the microglia themselves, which might remove a regulatory brake on microglia proliferation. The additional overexpression of CYLD in our model does not necessarily need to have the opposite effect, it rather seems likely that it would not have any additional benefit in this context. Furthermore, CYLD is crucial for maintaining neuronal activity, synaptic transmission and normal behavior of mice [14–17]. The loss of CYLD in microglia could contribute to these neurological phenotypes in *Cyld*^−/−^ mice, for example due to alterations in synaptic pruning conducted by the microglia. Nevertheless, it seems more likely that the absence of CYLD in neurons is the primary cause for these phenotypes, since CYLD highly accumulates in the postsynaptic density in a neuronal activity-dependent manner [34] and therefore can directly regulate synaptic signaling pathways.

In conclusion, overexpression of CYLD in microglia does not protect mice against neuroinflammation and thus is not a viable therapeutic strategy. Further studies using conditional CYLD knock-out mice are necessary to fully elucidate CYLD’s role in microglial regulation. Our novel Rosa26-Cyld-tdTomato mice provide a valuable tool for exploring CYLD’s function across various cell types, potentially advancing our understanding of inflammatory disorders and cancer.

## Methods

### Mice

All mice used were on a C57BL/6 background and housed under specific pathogen-free conditions. Rosa26-Cyld-tdTomato mice were generated by Taconic Biosciences. The genotypes of the generated Rosa26-Cyld-tdTomato mice were assessed with the following primers: forward_1 5’-TTGGGTCCACTCAGTAGATGC-3’; forward_2 5’- CTCTTCCCTCGTGATCTGCAACTCC-3’; reverse 5’-CATGTCTTTAATCTACCTCGATGG-3’. A PCR with all 3 primers detects the Rosa26 wildtype allele (299 bp; forward_2 and reverse primer) and the Rosa26-Cyld-tdTomato knock-in allele (744 bp; forward_1 and reverse primer). Rosa26-Cyld-tdTomato mice were crossed to Cx3cr1^CreER^ mice [23] to obtain R26-Cyld-OE^Cx3^ mice with the genotype Cx3Cr1^CreER/WT^; Rosa26^Cyld−OE−tdTom/WT^. Rosa26-tdRFP reporter mice [24] were crossed to R26-Cyld-OE^Cx3^ mice to obtain littermate control animals (R26^RFP/WT^) with the genotype Cx3Cr1^CreER/WT^; Rosa26^RFP/WT^. For the EAE experiment, heterozygous *Cyld*^+/-^ mice [25] were crossed to obtain *Cyld*^−/−^ mice and heterozygous (*Cyld*^+/-^) and homozygous (*Cyld*^+/+^) littermate controls.

### Tamoxifen treatment

Tamoxifen (Sigma-Aldrich) at a concentration of 20 mg/ml was dissolved in olive oil (Sigma-Aldrich) containing 5% ethanol by rotation overnight at 4 °C. Mice were injected subcutaneously with 100 μl (= 2 mg tamoxifen) at the age of 3–4 weeks on two days with a one-day break in between. R26^RFP/WT^ littermate controls were treated equally.

### Immunofluorescence stainings and Sholl analysis

Brains were isolated from mice perfused with 0.9% NaCl (Sigma-Aldrich), were fixed in 4% PFA for 6 h at 4 °C and cryoprotected in 30% sucrose. The tissue was embedded in TissueTek® O.C.T™ compound (Sakura Finetek) and 50 μm free-floating sections were cut at a cryostat.

Endogenous peroxidase was blocked in methanol containing 0.6% H_2_O_2_ for 20 min. at room temperature. Endogenous biotin and avidin binding sites were blocked with the Avidin/Biotin Blocking Kit (Vector Laboratories) for 15 min. each at room temperature. Sections were blocked with 10% ROTI®ImmunoBlock (Carl Roth) and 2% bovine serum albumin (BSA; PAN-Biotech) diluted in TBST (Tris Buffer Saline containing 1% TWEEN® 20) for 15 min. at room temperature. Sections were incubated with primary antibodies against Iba-1 (goat polyclonal, 1:500, FUJIFILM Wako Chemicals) and RFP (rabbit polyclonal, 1:1000, Rockland Immunochemicals) diluted in 2% BSA/TBST overnight at 4 °C. Sections were washed and incubated with secondary antibodies against goat IgG (donkey polyclonal, Alexa Fluor 647, 1:400, Invitrogen) and against rabbit IgG (donkey polyclonal, Biotin, 1:500, Invitrogen) in TBST for 1.5 h at room temperature. After washing, sections were treated with the TSA® Cyanine 3 detection kit (Akoya Biosciences) following manufacturer’s instructions. Sections were washed and mounted with Vectashield® Antifade Mounting Medium with DAPI (Vector Laboratories). For microglia quantification, images were acquired at a DMi8 widefield microscope (Leica) with an objective magnification of 20 × and only microglia with a clear cell body in-focus were manually counted. For Sholl analysis, images were acquired at a TCS SP8 multiphoton microscope (Leica) with an objective magnification of 20 × with 3 × optical zoom and 1.0 µm increments. Sholl analysis was performed in Fiji (ImageJ). First, Iba1 stacks were collapsed using maximum projection. The threshold was set to the same level for all images and only microglia with the cell body at the center of the stack analyzed. The cell body was defined and Sholl analysis was performed with a starting radius of 1 μm from the center and in increments of 1 μm.

### Cell isolation from the CNS

Brains were dissected from mice transcardially perfused with 0.9% NaCl solution (Sigma-Aldrich) and digested with 1 mg/ml Papain (Sigma-Aldrich) and 100 µg/ml DNase I (Roche) in HBSS with calcium and magnesium with mechanical dissociation with the GentleMACS homogenizer (Miltenyi, Bergisch Gladbach Germany) at 37 °C for 30 min. CNS homogenates were passed through a 70 μm cell strainer and cells were separated using a 70/30% Percoll (Sigma-Aldrich) gradient centrifugation for 45 min, 500 × *g* at 18 °C without brakes. Myelin was discarded and cells at the 70/30% interphase were carefully collected for further processing.

### Microglia isolation by magnetic-activated cell sorting (MACS)

Cells obtained from the CNS cell isolation were resuspended in 90 µl MACS buffer and 10 µl anti-CD11b MicroBeads (Miltenyi Biotec) and incubated for 15 min. at 4 °C. After washing, cells were applied to a MS column in an OctoMACS separator (Miltenyi Biotec), washed 3 × with MACS buffer and CD11b^+^ cells were eluted.

### Flow cytometry

Cells obtained from the CNS cell isolation were resuspended in anti-mouse CD16/CD32 (5 µg/ml, BioXCell,) in FACS buffer (PBS containing 2% fetal calf serum) for 10 min. at 4 °C to block Fc receptors. Cells were stained for 30 min at 4 °C on the cell surface with antibodies against CD4 BV421 (GK1.5, rat monoclonal, 1:400, BioLegend), CD45 BV510 (30-F11, rat monoclonal, 1:300, BioLegend), TCR-β FITC (H57-597, hamster monoclonal, 1:300, BioLegend), CD11b PECy7 (M1/70, rat monoclonal, 1:1000, eBioscience™, Thermo Fisher Scientific), CD8a PerCP (53–6.7, rat monoclonal, 1:500, BioLegend), MHC-II eFluor™450 (M5/114.15.2, rat monoclonal, 1:4000, eBioscience™, Thermo Fisher Scientific). For dead cell exclusion, fixable viability dye eFluor™780 (eBioscience™, Thermo Fisher Scientific) was added to the surface staining. Afterwards, cells were fixed and permeabilized with Cytofix/Cytoperm (BD Bioscience) and stained overnight at 4 °C with anti-CD68 APC antibody (FA-11, 1:500, rat monoclonal, BioLegend). Samples were acquired at a FACSCanto II using FACS Diva software (BD Bioscience) and analyzed with FlowJoTM v10 software (BD Bioscience). For all analyses, doublets (FSC and SSC properties) and dead cells were excluded.

### RNA isolation, reverse transcription and real-time PCR

RNA was isolated with the ReliaPrep™ RNA Cell Miniprep System (Promega) following manufacturer’s guidelines. RNA concentrations were determined by measuring absorbance at 260 nm and 280 nm using the NanoQuant Plate™ (Tecan) at an Infinite M200 pro plate reader (Tecan). cDNA was synthesized with the QuantiTect® Reverse Transcription Kit (Qiagen) using all RNA obtained following manufacturer’s guidelines. Real-time PCR (RT-PCR) was performed with the StepOnePlus™ Real-Time PCR System (Life Technologies) using SYBR Green reagent (Promega). Fold enrichment was calculated using the Delta-Delta CT method normalized to hypoxanthin-guanin-phosphoribosyltransferase (*Hprt*) as house-keeping gene reference. *Hprt* primers were self-designed: forward 5’-CGTCGTGATTAGCGATGATG-3’, reverse 5’- TCCAAATCCTCGGCATAATG-3’. *Cyld* primers were ordered as QuantiTect Primer Assay (QT00103768, Qiagen).

### RNA isolation and RNA sequencing

For RNA sequencing, RNA was isolated with the RNeasy Micro Kit (Qiagen). RNA quality was assessed with the Qubit RNA High Sensitivity Assay Kit on a Qubit 3 Fluorometer (Invitrogen) and with the High Sensitivity RNA ScreenTape on a 4200 TapeStation system (Agilent). 100 ng RNA were used for library preparation with the NEBNext Single Cell/Low Input RNA Library Prep Kit for Illumina using NEBNext Multiplex Oligos for Illumina (New England Biolabs). cDNA quality was assessed with Agilent’s High Sensitivity DNA kit on an Agilent 2100 Bioanalyzer. Quantity of the library was assessed with the Qubit 1X dsDNA assay kit on a Qubit 4 Fluorometer (Invitrogen) and library size was determined with a D1000 ScreenTape analysis on a 4200 TapeStation system (Agilent). All libraries were sequenced in paired-end mode (2 × 50 nt) on an Illumina NovaSeq 6000 instrument resulting in an average of 20 million distinct sequencing reads per library.

### RNA sequencing data analysis

Quality control of the sequencing data was performed with the FastQC tool (v0.12.1). RNA sequencing reads were mapped to the Mus_musculus.GRCm38 reference genome (ENSEMBL v84) using kallisto (v0.46.0). Alignments were processed with kallisto to obtain gene transcripts per million (TPM) values and gene counts. Differential gene expression analysis was performed with DESeq2 (v1.34.0) and apeglm (v1.16.0) was applied for shrinkage of log-fold changes.

### Experimental autoimmune encephalomyelitis (EAE)

Mice were immunized 12 weeks after tamoxifen administration at the age of 15–16 weeks. MOG_35-55_/CFA was prepared by mixing 1 mg/ml MOG_35-55_ (GenScript) in PBS with Complete Freund’s Adjuvants (CFA, BD Biosciences) containing *mycobacterium tuberculosis* H37RA (BD Biosciences). 50 μg of the MOG_35-55_/CFA emulsion were injected subcutaneously into the tail base. Mice were also injected intraperitoneally with 150 ng pertussis toxin (List Biological Laboratories) in PBS at the day of immunization and two days later.

Mice were weighed daily and clinical scores were documented as follows: 0 - no disease; 0.5 - limb tail; 1 - paralyzed tail; 1.5 - weakened righting reflex; 2 - no righting reflex; 3 - partial paralysis of hind legs; 3.5 - paralysis of one hind leg; 4 - paralysis of both hind legs.

### LPS-induced neuroinflammation

LPS (from *Salmonella typhimurium*, Sigma-Aldrich) was solved in PBS at a concentration of 0.2 mg/ml. Mice were injected 12 weeks after tamoxifen administration at the age of 15–16 weeks with 1 mg/kg LPS intraperitoneally and were sacrificed 24 h later.

### Statistics

Statistical analyses were performed with Prism v10 software (GraphPad). Values are shown as mean ± standard error of the mean (SEM) unless indicated otherwise. *P* values were considered significant with * p < 0.05, ** p < 0.01, *** p < 0.001 and **** p < 0.0001.

## Data Availability

The RNA sequencing data generated in this study will be made available on request.
